# Genetic assessment of litter size, body weight, carcass traits and gene expression profiles in exotic and indigenous rabbit breeds: a study on New Zealand White, Californian, and Gabali rabbits in Egypt

**DOI:** 10.1007/s11250-024-04082-z

**Published:** 2024-08-22

**Authors:** Mohamed S. Ayyat, Usama M. Abd El-Monem, Mahmoud M. A. Moustafa, Adham A. Al-Sagheer, Mohamed D. Mahran, Mahmoud M. El-Attrouny

**Affiliations:** 1 Department of Animal Production, Faculty of Agriculture, Zagazig University, Zagazig, 44511 Egypt; 2 Department of Genetics and Genetic Engineering, Faculty of Agriculture, Benha University, Moshtohor, Toukh, 13736 Egypt; 3 Department of Animal Production, Faculty of Agriculture, Benha University, Moshtohor, Toukh, 13736 Egypt

**Keywords:** Rabbits, Litter traits, heritability, Correlation, Gene expression profile

## Abstract

Rabbits are essential for commercial meat production due to their efficient growth and productivity, breeds like New Zealand White (NZW), Californian (CAL), and Gabali (GAB) rabbits offer unique genetic traits in litter, growth, and carcass traits. This study aimed to evaluate heritability (h^2^), genetic and phenotypic correlations (rg and rp) for litter size, body weight and carcass traits across California (CAL), New Zealand white (NZW) and Gabali (GA) rabbits. Along with exploring gene expression profiles of *TBC1D1*, *NPY*, *AGRP*, *POMC*, Leptin, *GH*, *GHR*, *IGF-1*, *CAA*, *GPR*, *ACC*, *CPT1*, *FAS*, and *CART* in the brain, liver, and meat tissues of different rabbit breeds. The breed genotype had a significant impact on litter size (LS), litter weight (LW), body weight at 12 weeks (BW12), and daily weight gain (DWG) traits. NZW rabbits displayed superior performance in terms of litter size and litter weight, while CAL rabbits recorded the highest values for BW12 and DWG. Heritability estimates (h^2^) were generally low for litter size (ranging from 0.05 to 0.12) and medium for body weight (ranging from 0.16 to 0.31). Both genetic (r_g_) and phenotypic (r_p_) correlations for litter size were positive and moderate (ranging from 0.08 to 0.48), while correlations for body weight ranged from 0.21 to 0.58. Additionally, CAL rabbits exhibited higher carcass traits compared to NZW and GA rabbits. In terms of breed-specific gene expression patterns, New Zealand White (NZW) rabbits displayed the highest expression levels of key genes related to energy metabolism (*TBC1D1*), appetite regulation (*NPY*,* AGRP*,* POMC*), nutrient transport (*CAA*), and G protein-coupled receptors (*GPR*) in both brain and liver tissues. Californian *(CAL*) rabbits exhibited superior gene expression of the *ACC* gene in brain tissue and *GH*,* GHR*, and *IGF-1* genes in brain and meat tissues. Gabali (GAB) rabbits demonstrated the highest expression levels of *TBC1D1*,* NPY*,* AGRP*,* GPR*, and *ACC* genes in meat tissues. These breed-specific gene expression differences, combined with genetic evaluation efforts, have the potential to enhance reproductive and productive performance in rabbits, offering valuable insights for rabbit breeding programs and genetic selection.

## Introduction

Rabbits exhibit several advantageous traits, such as small size, rapid growth, and the production of protein-rich, low-fat meat. They have a short generation interval, require minimal space, show high conception rates, reach sexual maturity quickly, and have a short gestation period. These factors contribute to their high productivity and ease of rearing, enhancing the profitability of rabbit farming, particularly in developing countries (Lukefahr et al. 2022; Qodirova and Ruzikulova 2023; Erdaw and Beyene 2022; Parlasca and Qaim 2022). Genetic and environmental factors play crucial roles in influencing the reproductive performance of rabbit breeds (Apori et al. 2015).

Among rabbit breeds, New Zealand White (NZW), Californian (CAL), and Gabali (GAB) are essential for rabbit farming due to their unique traits. NZW rabbits excel in fertility, growth, and feed efficiency, making them ideal for meat production (Wanjala et al. 2015). CAL rabbits are known for their meat quality and adaptability to various climates (Kumar et al. 2023a). GAB rabbits, native to Egypt, thrive in arid climates and are resistant to local diseases, supporting sustainable meat production in challenging environments (Badr et al. 2019). Together, these breeds offer a diverse genetic foundation crucial for breeding programs aimed at enhancing global productivity and meat quality.

In commercial rabbit farming, body weight, litter, growth, and carcass traits are crucial indicators of economic success (García and Argente 2021). Calculating genetic parameters such as heritability (h^2^), phenotypic, and genetic correlations (rp&rg) for these characteristics is necessary for conducting breeding programs to increase the productivity of the rabbits. (Elfadil et al. 2023; Peiró et al. 2021). However, research on genetic parameters related to growth and carcass traits remains limited (Blasco et al. 2018). Hence, there is a pressing need for further research to elucidate these genetic parameters essential for effective improvement programs.

Integrating the genetic parameter estimates of these economic traits with their molecular-level gene expression profiles across different breeds holds promise for advancing breeding programs and enhancing productivity in rabbit industry (Ezzeroug et al. 2019). Therefore, we aimed to evaluate heritability (h^2^), genetic and phenotypic correlations (r_g_ and r_p_) for litter size, body weight and carcass traits of CAL and NZW breeds, both of foreign origin and the GA breed, a local breed. Additionally, gene expression profiles of *TBC1D1*,* NPY*,* AGRP*,* POMC*,* Leptin*,* GH*,* GHR*,* IGF-1*,* MC1R*,* GPR*,* ACC*,* CPT1*,* FAS*, and *CART* in the hepatic, brain, and meat tissues of these breeds were investigated to enhance our understanding of the genetic regulation of these economic traits. By correlating molecular data with genetic parameter estimates, we aim to develop effective breeding programs to meet the growing global demand for high-quality rabbit meat.

## Materials and methods

### Animals, population structure, housing, and feeding

The experimental protocols applied in this study were performed in accordance with the guidelines for animal welfare of the Animal Production Department of Zagazig University, Egypt, and were approved by the Ethics Committee of the Local Experimental Animals Care with the assigned approval number ZU-IACU/2/F/100/2018. Every effort was made to minimize the suffering of the animals involved.

This study used two foreign breeds, the Californian (CAL) and the New Zealand White (NZW), along with the Sinai Gabali breed (GAB). Information from 105 does and 37 bucks (26 female, 11 male GAB), (48 female, 18 male NZW), and (31 female, 11 male CAL). At four weeks of age, the litters were weaned. The rabbitry was housed in a semi-confined space. Breeding does and sires were housed separately in standard-sized wire cages of 50 × 50 × 30 cm^3^. All of the animals in the rabbitry were housed in climate-controlled environments, which included 16 h of light and 8 h of darkness, a range of ambient temperatures from 22 to 30 degrees Celsius, and relative humidity from 24 to 50%. Natural mating was used to mate each buck with three does. Male and female rabbits were initially mated at 4.5 to 5 months of age, and subsequent mating was conducted ten days after delivery. For mating purposes, does were placed in the buck’s cage and then singly recorded. Avoiding sire and daughter mattings, as well as full and half sibs. After 10 days of service, the mated doe was palpated to check for pregnancy. On test day, the negative does were returned to the same buck to be rebred. After 25 days of conception, a metal nest box containing a thin layer of rice straw was prepared for each pregnant doe. Within 24 h of giving birth, the litters were carefully inspected, weighed, and recorded. When the bunnies reached 28 days old (weaning age), they were separated from their mothers’ cages, weighed, tagged with ear tags, and placed in communal cages with automatic water nipples.

A commercial diet in pellet form was provided to growing rabbits; the contents of the pelleted diet were crude protein, ether extract, nitrogen-free extract, crude fiber, and ash (17.9%, 2.45%, 58.5%, 15.52%, and 6.29%, respectively). While breeding rabbits were fed a pelleted diet consisting of 13%, 2.54%, and 17.4% of crude fiber, ether extract, and crude protein, on a dry matter basis, respectively (De Blas and Mateos 2010) over the entire study period. Ad libitum access to feed and water was provided. Breeding and growing rabbits were housed in identical environments, with consistent sanitation and management practices.

### Data collection

Data were meticulously collected throughout the study period for several traits. Litter characteristics examined included number born alive (NBA), litter weight at birth (LWB), at 21 day (LW21) and at wean (LWW). Litter size at birth (LSB), at weaning (LSW), and at 21 days (LS21) were also included. Following weaning, 906 animals had their body weight (BW) measured at 4 (BW4), 8 (BW8), and 12 (BW12) weeks of age. Daily gain (DG) was calculated at intervals of 4 (DG4-8), 4 (12) (DG4-12), and 8 (12) (DG8-12) weeks of age.

Upon completion of the experiment, ten rabbits from each breed, consisting of five females and five males at 12 weeks of age experienced a fasting period of approximately 12 h before being weighed and subsequently slaughtered. The slaughtering process encompassed bleeding and skinning the rabbits. After the rabbits were slaughtered, the weight of their skin was measured directly from the neck to the base of the tail. After evisceration of the skinned carcasses, the weight of internal organs (heart, kidneys, and liver) as well as carcass by-products was measured. Subsequently, the carcasses were divided into four sections and assessed according to the procedure established by the World Rabbit Science Association (WRSA) (Blasco et al. 1993). The four sections of the carcasses were weighed: the foreleg weight section (FLW) located between the atlas and the twelfth thoracic vertebra; the intermediate section (TW) positioned between the seventh lumbar vertebra and the twelfth thoracic vertebra; the loin weight (LW); and the hind leg weight part (HLW) extending from the seventh lumbar vertebrae.

### Gene expression profiles

Examining gene expression profiles across different tissues allows breeders to understand the regulatory mechanisms governing trait expression. This knowledge enables breeders to manipulate gene expression through selective breeding, genetic engineering, or management practices to optimize desired traits in rabbit populations. Therefore, in the current study and using the GenezolTM TriRNA Pure Kit (GZX050, GZXD050), total RNA was extracted from the brain, liver, and meat (5 samples/tissue/ breed). Each sample’s 50 mg of unfrozen tissue was ground using liquid nitrogen. Using a tube (2 mL) containing 700 µL of GENEzolTM Reagent, the tissue was homogenized. Following homogenization, the sample was transferred to a 2 mL RNase-free tube and centrifuged at 14,000 rpm for one minute. After adding the same amount of molecular-grade absolute ethanol, the mixture was mixed thoroughly. To extract the follow-through, the RB column was then put in a different 2 mL collection tube and centrifuged at 14,000 rpm for one minute. Once the RB column is ready, repeat these steps. Another sanitized 2 mL vial held the prepared RB column. 50 µL of freshly made DNase I was added to the center of the column, and then 400 µL of pre-wash buffer was added to pre-wash the column. The resulting follow-through material was disposed of after the pre-wash buffer was spun for a minute at 14,000 rpm. After centrifuging the column for three minutes, it was further cleaned. Using 50 µL of RNase-free water, the RNA was extracted from the column and promptly kept at -80 °C in preparation for the RT-PCR that followed, as per (Brunt 2000) instructions. The concentration and purity of RNA samples were determined using a Nano-Drop 2000 C spectrophotometer (Thermo Scientific, USA). The absorbance ratio (A260/A280) for all samples was found to be 2.00 ± 0.10, indicating good purity. The integrity of the RNA was assessed by visualizing the samples on a 2% agarose gel using gel electrophoresis and imaged with a Gel Doc (BioRad).

Two steps were involved in the cDNA process, which used 20 µL. In the first, 1.5 µL of RNase-free water, 2 µL of oligo (dt) 14 primers (1 µM) (Table 1), and 10 µL of total RNA were incubated for 10 min in a PCR machine (Senso Quest, Hilden, Germany). The second step involved preparing and adding to the first step 4 µL of a 5X first strand buffer, 0.5 µL of H minus MMLV (200 unit/µL), and 2 µL of dNTPs combination (10 mM). Then, for sixty minutes, this mixture was maintained at 42 °C. For the qPCR processes that followed, the original cDNA samples were kept at -80 °C in accordance with the technique outlined by (AlGeffari et al. 2023).

**Table 1:** Primers used in PCR analysis

Gene	Primer sequence (5′–3′)		Reference
*GAPDH*	F	*TGCCACCCACTCCTCTACCTTCG*	(Liu et al. 2017)
	R	CCGGTGGTTTGAGGGCTCTTACT	
*NPY*	F	CCTCATCACCAGGCAGAGAT	(Liu et al. 2017)
	R	ATTTCGTTTCCCATCACCAC	
*AgRP*	F	GCTACTGCCGCTTCTTCAAC	(Liu et al. 2017)
	R	CCATTCTTTATTGGCGTTCC	
*POMC*	F	GCCTGGAAGATGCTGAGGT	(Liu et al. 2017)
	R	CTCCTGACACTGGCTGCTCT	
*CART*	F	AGGAGCCAGGATTGGGAAG	(Liu et al. 2017)
	R	CTGATGGAAGAGCGTGGAAG	
*GPR43*	F	CGTCCAACTTCCGCTGGTA	(Liu et al. 2017)
	R	CTTGTACTGCACGGGGTAGG	
*ACC*	F	GTGGTCTTCGTGTGAACTGG	(Liu et al. 2017)
	R	TTCTTCTGCTGCCTTTAGCC	
*FAS*	F	ACCACGTCCAAGGAGAGCA	(Liu et al. 2017)
	R	AGTTCTGCACCGAGTTGAG	
*CPT1*	F	ATTCTCACCGCTTTGGGAGG	(Liu et al. 2017)
	R	ACGGGGTTTTCTAGGAGCA	
*IGF-1*	F	ACCGCAACTACCGCTTCCCC	(Feng and von Bartheld 2011)
	R	CCGCGGATGACCGTGAGGTT	
*Leptin*	F	CACACGCAGTCGGTCTCCT	(Koch et al. 2013)
	R	GTTTGGACTTCATCCCTGGC	
*GHR*	F	AATCCACCTTCAACC CTA TC	(Sahwan et al. 2014)
	R	CGGAGA CTT CTTACA ATGGC	
*GH*	F	CTCCAGGGCTAGAAGGGAAC	(El-Sabrout and Aggag 2017)
	R	CTCACTTCTGCGCTCAATCC	
*TBC1D1*	F	TTCCAGAAAGGAGCCCGTGAC	(Yang et al. 2013)
	R	GGTTGACTCTTGCCCAGGT	
*MC1R*	F	GGGACTATGCCCATGCAG	(Fontanesi et al. 2010)
	R	CCACTACCAGCAGGTTCTCC	

*GAPDH*, glyceraldehyde 3-phosphate dehydrogenase; *NPY*, neuropeptide Y; *AgRP*, agouti-related protein; *POMC*, pro-opiomelanocortin; *CART*, cocaine-amphetamine-regulated transcript; *GPR43*, G-protein-coupled receptor 43; *ACC*, acetyl-CoA carboxylase alpha; *FAS*, fatty acid synthase; *CPT1*, carnitine palmitoyltransferase 1; *IGF-1*, insulin-like growth factor 1; *GHR*, growth hormone receptor; *GH*, growth hormone; *TBC1D1*; TBC1, domain family member 1; *MC1R*, melanocortin 1 receptor

RT-qPCR was conducted for each sample, with three biological replicates. A total of 25 µL of nuclease-free water, 1 µM of each forward and reverse primer, 10 nM, 0.4 µL ROX Dye (50X), 10 µL of mater mix (A.B.T.TM 2X qPCR SYBR-Green MasterMix, ROX, Q03-02-01), and 200 ng of cDNA were used in each qPCR reaction. Using an applicable Biosystem Real-Time PCR System, the reactions were examined under the following two-step cycle conditions: After ten minutes at 95 °C, there are forty cycles of 95 °C for fifteen seconds and 60 °C for sixty seconds. The quantification of target genes was normalized using the housekeeping gene glyceraldehyde-3-phosphate dehydrogenase (*GAPDH*), a well-established reference gene in rabbit-related research (Sobajima et al. 2005; Zhang et al. 2019). The mRNA levels of target and reference genes were determined using absolute quantification of their CT values. The mRNA relative expression level was calculated by dividing the copy concentration of target genes (CT values) by that of the endogenous reference (CT of target gene/CT of *GAPDH*) (El-Attrouny et al. 2021; Lu et al. 2008).

### Statistical analysis

Descriptive statistics for performance traits, including (LS, LW, BW, DG, and carcass traits) were computed using the UNIVARIATE procedure in the SAS software (SAS 2002). Mean differences were evaluated using Duncan’s multiple range test, with significance set at *p* < 0.05 (Duncan 1955). The statistical model has the following form; Yij = µ + B_i_ + ɛ_ij_. Where, Y_ij_ represents the individual observation for each characteristic, µ denotes the overall mean, B_i_ represents the breed’s fixed effect (i = 1…0.3), and ɛ_ij_ represents the random residual effect ~ NID (0, σ^2^e).

The following multi-trait animal model was used, y = Xb + Z_a_U_a_ + e. In this model, y represents the vector of observations for litter traits, body weight, and carcass traits. The design matrix for fixed effects (X) includes the breed effects as well as any other relevant fixed effects. The vector b represents the corresponding fixed effect coefficients. Za represents the design matrix for the random additive genetic effects, and Ua represents the vector of additive genetic effects. The variance components of random effects, heritabilities (h^2^), and genetic and phenotypic correlations (r_g_&r_p_) among all trait combinations were estimated using the VCE6 software (Groeneveld et al. 2010), Heritabilities for body weight and litter traits were computed as:

$$h_{\,\,\,a}^2 = {{\sigma _{\,\,\,a}^2} \over {\sigma _{\,\,\,{\rm{a}}}^2 + \sigma _{\,\,\,{\rm{e}}}^2}}$$

Where: σ^2^_a_ and σ^2^_e_ are the variances due to the effects of direct additive genetic and random error, respectively.

The genetic (*r*_*g*_) and phenotypic (*r*_*p*_) correlations among body weight and litter traits were estimated according to the formula of Quaas et al. (1984) and Becker (1984):

$${r_g} = {{{\mathop{\rm cov}} \,(x)_{ij}} \over {\sqrt {{\mathop{\rm var}} \,(x)_{ii} \cdot \,{\mathop{\rm var}} \,(x)} _{jj}}}$$

$${r_p} = {{{{{\mathop{\rm cov}} }_e} + {{{\mathop{\rm cov}} }_a}} \over \matrix{\sqrt {\left[ {{\sigma ^2}{e_{(X1)}} + {\sigma ^2}{a_{(X1)}}} \right]} \hfill \cr + \left[ {{\sigma ^2}{e_{(X2)}} + {\sigma ^2}{a_{(X2)}}} \right] \hfill \cr} }$$

Where: Cov (X)_ij_ = the covariances among additive genetic effects for body weight and litter traits; X_ii_ and X_jj_ = the additive genetic (a) variances of i^th^ and j^th^ all traits. Cov_e_ = covariance of error among all traits; Cov_h_ = covariance among all traits for the rabbit ; σ^2^e_(X1)_ = the variance of error for trait 1; σ^2^a_(X1)_ = the additive variance of rabbit for trait 1; σ^2^e_(X2)_ = the variance of error for trait 2; σ^2^a_(X2)_ = the additive variance of rabbit for trait 2.

For gene expression data, Data were analyzed for each breed in different tissues using (SAS 2002). Differences among breeds were considered significant at *p* ≤ 0.05 and trending where at (0.05 > *p* ≤ 0.1). Significant differences between means were tested by Duncan’s multiple range test (Duncan 1955).

## Results

### Litter and growth traits

The analysis of litter traits, as presented in Table 2, highlighted significant breed effects (*P* < 0.001), indicating distinct reproductive performances among New Zealand White (NZW), Californian (CAL), and Gabali (GAB) rabbit breeds. Notably, NZW rabbits exhibited superiority across all litter traits, including the number of kits born alive (NBA), litter size at birth (LSB), litter size at 21 days (LS21), and litter size at weaning (LSW). Conversely, GAB rabbits consistently recorded the lowest values for these litter traits compared to NZW and CAL rabbits. Additionally, CAL rabbits displayed the highest litter weight at birth (LWB), while NZW rabbits showed the highest litter weight at 21 days (LW21) and litter weight at weaning (LWW), indicating breed-specific variations in reproductive performance.

**Table 2:** Least squares mean and standard errors (SE) for litter and body weight traits in three different rabbit breeds

Items	Breed			*p*-value
	NZW	CAL	GAB	
**Litter traits**				
NBA (kids)	6.80 ± 0.18^a^	6.68 ± 0.34^a^	5.32 ± 0.41^b^	0.0055
LSB (kids)	7.00 ± 0.18^a^	6.92 ± 0.33^a^	5.15 ± 0.38^b^	< 0.0001
LS21(kids)	6.33 ± 0.19^a^	5.68 ± 0.33^a^	4.21 ± 0.43^b^	< 0.0001
LSW (kids)	6.10 ± 0.18^a^	5.35 ± 0.32^a^	3.83 ± 0.41^b^	< 0.0001
LWB (g)	346.95 ± 9.86^ab^	361.80 ± 17.90^a^	302.14 ± 21.66^b^	0.0928
LW21(g)	1776.5 ± 56.68^a^	1548 ± 0.54^ab^	1368.0 ± 45.26^b^	0.0050
LWW (g)	2575.5 ± 54.32^a^	2333.0 ± 55.12^ab^	1822.6 ± 43.22b	0.0002
**Body Weight at**				
BW4	433.05 ± 4.52 ^a^	430.12 ± 9.48 ^a^	443.60 ± 13.74 ^a^	0.7313
BW8	872.31 ± 5.20 ^a^	881.80 ± 13.24 ^a^	874.76 ± 17.79 ^a^	0.8096
BW12	1836.56 ± 7.24^a^	1854.32 ± 14.86^a^	1782.16 ± 19.94^b^	0.0131
**Daily Gain (g)**				
DG 4–8	15.32 ± 0.19 ^a^	15.69 ± 0.41 ^a^	15.25 ± 0.56 ^a^	0.6999
DG 8–12	34.09 ± 0.19^a^	34.66 ± 0.41^a^	32.34 ± 0.56^b^	0.0158
DG 4–12	24.83 ± 0.13^a^	25.24 ± 0.27^a^	23.81 ± 0.36^b^	0.0069

NZW, New Zealand White; CAL, Californian; GAB, Gabali. NBA, number born alive; LSB, litter size at birth; LWB, litter weight at birth; LS21d, litter size at 21 days; LW21d, litter weight at 21 days; LSW, litter size at weaning; LWW, litter weight at weaning. BW4, BW8, and BW12, body weight at 4, 8, and 12 weeks of age, respectively; DG4-8, DG8-12and DG4-12, daily gain during the intervals from 4 to 8, 8 to 12 and 4 to 12 weeks of age, respectively.

^a, b^ Means in the same row with different letters are significantly different at *P* < 0.05

Moving to body weight (BW) and daily gain (DG) analysis, it was observed that both traits increased with advancing age, reflecting growth progression in all rabbit breeds. Significant breed effects (*P* < 0.05) were evident for BW at 12 weeks and DG at 8–12 and 4–12 weeks. CAL rabbits exhibited the highest BW at 12 weeks and DG at 8–12 and 4–12 weeks, although the differences were not statistically significant from NZW rabbits, suggesting similarities in growth patterns between these two breeds.

### Genetic parameters

#### Heritability (h^2^)

The heritability (h^2^) estimates, detailed in Table 3, varied across different traits, indicating the degree of genetic influence on reproductive and growth-related traits. Moderate to high h^2^ values were observed for litter traits, ranging from 0.05 for LSB to 0.31 for DG 8–12 weeks, suggesting significant genetic components in reproductive performance. Similarly, moderate h^2^ values were noted for BW and DG traits, ranging from 0.16 for BW4 to 0.31 for DG 8–12, emphasizing the genetic potential for growth improvement through selective breeding.

**Table 3:** Estimates of heritability ± S.E (on the diagonal), genetic correlation (above the diagonal), phenotypic correlation (below the diagonal), and the associated standard errors for litter traits of rabbits

Traits	NBA	LSB	LWB	LS21	LW21	LSW	LWW
**NBA**	**0.06 ± 0.01**	0.39 ± 0.06	0.43 ± 0.04	0.19 ± 0.02	0.21 ± 0.03	0.09 ± 0.02	0.17 ± 0.03
**LSB**	0.28 ± 0.02	**0.05 ± 0.02**	0.35 ± 0.02	0.27 ± 0.03	0.26 ± 0.04	0.37 ± 0.02	0.16 ± 0.04
**LWB**	0.48 ± 0.07	0.24 ± 0.03	**0.12 ± 0.03**	0.14 ± 0.04	0.41 ± 0.07	0.08 ± 0.02	0.44 ± 0.08
**LS21**	0.22 ± 0.02	0.21 ± 0.04	0.08 ± 0.01	**0.09 ± 0.02**	0.39 ± 0.03	0.13 ± 0.01	0.25 ± 0.04
**LW21**	0.19 ± 0.01	0.32 ± 0.07	0.28 ± 0.02	0.41 ± 0.09	**0.07 ± 0.02**	0.10 ± 0.02	0.43 ± 0.08
**LSW**	0.11 ± 0.01	0.27 ± 0.04	0.18 ± 0.02	0.34 ± 0.04	0.19 ± 0.02	**0.05 ± 0.01**	0.33 ± 0.04
**LWW**	0.13 ± 0.02	0.24 ± 0.03	0.39 ± 0.04	0.31 ± 0.05	0.29 ± 0.03	0.43 ± 0.07	**0.11 ± 0.04**

NBA, number born alive; LSB, litter size at birth; LWB, litter weight at birth; LS21, litter size at 21 days; LW21, litter weight at 21 days; LSW, Litter size at weaning; LWW, litter weight at weaning

### Phenotypic and genetic correlations (r_p_&r_g_)

Genetic and phenotypic correlations among traits, as depicted in Tables 3 and 4, provided insights into the relationships between different reproductive and growth-related parameters. Moderate positive genetic correlations (r_g_) were observed among litter traits, indicating shared genetic factors influencing reproductive performance. Positive r_g_ values were also evident between litter traits and BW at different ages, highlighting the interconnectedness of these traits at the genetic level. Phenotypic correlations (r_p_) between litter traits and between BW and DG at different ages were positive and often high to moderate, underscoring significant phenotypic associations among these traits, which is crucial for effective breeding strategies.

**Table 4:** Estimates of heritability ± S.E (on the diagonal), genetic correlation (above the diagonal), and phenotypic correlation (below the diagonal) for body weight and daily gain of rabbits

Traits	BW4	BW8	BW12	DG4-8	DG8-12	DG4-12
BW4	**0.16 ± 0.02**	0.34 ± 0.05	0.47 ± 0.07	0.29 ± 0.04	0.31 ± 0.05	0.39 ± 0.06
BW8	0.52 ± 0.05	**0.29 ± 0.05**	0.42 ± 0.06	0.36 ± 0.05	0.24 ± 0.04	0.30 ± 0.05
BW12	0.43 ± 0.06	0.54 ± 0.09	**0.23 ± 0.05**	0.22 ± 0.04	0.37 ± 0.05	0.25 ± 0.03
DG4-8	0.38 ± 0.05	0.49 ± 0.04	0.38 ± 0.06	**0.20 ± 0.04**	0.21 ± 0.03	0.32 ± 0.05
DG8-12	0.44 ± 0.07	0.21 ± 0.03	0.42 ± 0.07	0.37 ± 0.04	**0.31 ± 0.07**	0.41 ± 0.06
DG4-12	0.51 ± 0.10	0.42 ± 0.07	0.39 ± 0.04	0.41 ± 0.8	0.58 ± 0.11	**0.26 ± 0.06**

BW4, BW8, and BW12, body weight at 4, 8, and 12 weeks of age, respectively; DG4-8, DG8-12, and DG4-12, daily gain during the intervals from 4 to 8, 8 to 12 and 4 to 12 weeks of age, respectively

### Carcass traits

The analysis of carcass traits, detailed in Table 5, revealed significant breed effects on various carcass characteristics. CAL rabbits generally exhibited higher values compared to NZW and GAB rabbits, particularly in slaughter weight, full gastrointestinal tract weight, hot carcass weight, and other carcass traits, indicating breed-specific differences in carcass composition and meat yield.

**Table 5:** Least squares mean and standard errors (SE) for slaughter and carcass traits in three different rabbit breeds

Items	Breed			*p*-value
	NZW	CAL	GAB	
SW (g)	1781.80 ± 50.5^ab^	1918.80 ± 50.5^a^	1679.00 ± 50.5^b^	0.0185
CSkW (g)	312.60 ± 8.26^b^	340.80 ± 8.26^a^	306.0 ± 8.26^b^	0.0263
FGTW (g)	375.40 ± 11.53^a^	347.20 ± 11.53^a^	290.0 ± 11.53^b^	0.0007
HCW (g)	1063.80 ± 34.13^ab^	1144.60 ± 34.13^a^	1008.60 ± 34.13^b^	0.0462
HW (g)	105.80 ± 2.02^a^	107.60 ± 2.02^a^	97.00 ± 2.02^b^	0.0066
LvW (g)	51.68 ± 4.91 ^a^	52.07 ± 4.91 ^a^	55.35 ± 4.91 ^a^	0.8472
KiW (g)	13.06 ± 1.29 ^a^	12.46 ± 1.29 ^a^	11.05 ± 1.29 ^a^	0.5509
LHW (g)	18.58 ± 1.47 ^a^	19.46 ± 1.47 ^a^	14.98 ± 1.47 ^a^	0.1176
HLW (g)	387.20 ± 11.62^a^	400.80 ± 11.62^a^	343.20 ± 11.62^b^	0.0111
LW (g)	159.80 ± 8.21 ^a^	162.80 ± 8.21 ^a^	147.20 ± 8.21 ^a^	0.3910
FLW (g)	249.80 ± 9.61	262.20 ± 9.61	241.40 ± 9.61	0.3393
SFaW (g)	0.98 ± 0.24^b^	1.85 ± 0.21^a^	1.08 ± 0.21^b^	0.0366
PFaW (g)	2.41 ± 0.71 ^a^	2.41 ± 0.63 ^a^	1.77 ± 0.63 ^a^	0.7319
HW (g)	5.11 ± 0.38 ^a^	5.32 ± 0.34 ^a^	4.99 ± 0.34 ^a^	0.7982
LW (g)	13.19 ± 1.29 ^a^	13.41 ± 1.15 ^a^	9.18 ± 1.15 ^a^	0.0943

^a, b^ Means in the same row with different letters are significantly different at *P* < 0.05

SW, Slaughter weight; CSkW, commercial skin weight; FGTW, full gastrointestinal tract weight; HCW, hot carcass weight; HW, head weight; LvW, liver weight; KiW, kidneys weight; LHW, thoracic viscera weight; HLW, hind leg weight; LW, loin weight; FLW, for leg weight; SFaW, scapular fat weight; PFaW, perirenal fat weight; HW, Heart weight; LW, Lung weight

### Gene expression profiles

**Fig. 1:**
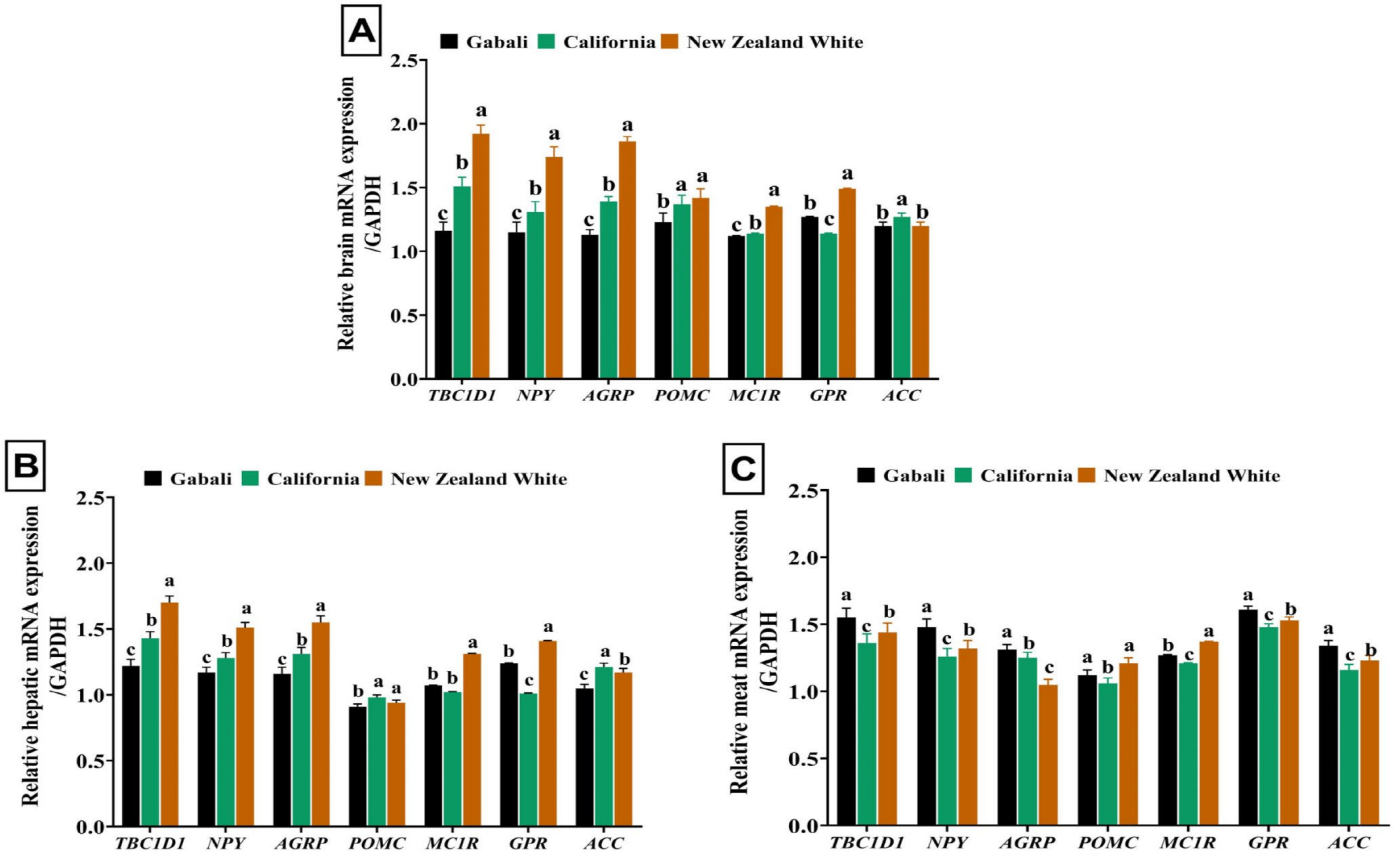
Gene expression profile of *TBC1D1*,* NPY*,* AGRP*,* POMC*,* MC1R*,* GPR*, and *ACC* genes in brain (**A**), liver (**B**), and meat (**C**) tissues of California, New Zealand White, and Gabali rabbits. *ACC*, acetyl-CoA carboxylase alpha gene; *AgRP*, agouti-related protein gene; *GPR43*, G-protein-coupled receptor 43 gene; *MC1R*, melanocortin 1 receptor gene; *NPY*, neuropeptide Y gene; *POMC*, pro-opiomelanocortin gene; *TBC1D1*; TBC1, domain family member 1 gene

The gene expression profiles in different tissues revealed notable variations among New Zealand White (NZW), Californian (CAL), and Gabali (GAB) rabbit breeds. In brain tissue (Fig. 1), NZW rabbits exhibited the highest expression levels of genes associated with energy metabolism (*TBC1D1*), appetite regulation (*NPY*, *AGRP*, *POMC*), melanocortin receptor (*MC1R*), and G protein-coupled receptors (*GPR*), surpassing CAL and GAB rabbits. Conversely, CAL rabbits displayed the highest expression profile for the *ACC* gene in brain tissue. This pattern was consistent in liver tissue, where NZW rabbits generally exhibited superior gene expression levels across all genes except for POMC and ACC, which were highest in CAL rabbits. Interestingly, GAB rabbits demonstrated the highest gene expression profiles for several genes in meat tissues, including *TBC1D1*, *NPY*, *AGRP*, *GPR*, and * ACC*, although *POMC* and *MC1R* exhibited the highest upregulated profiles in NZW rabbits.

**Fig. 2:**
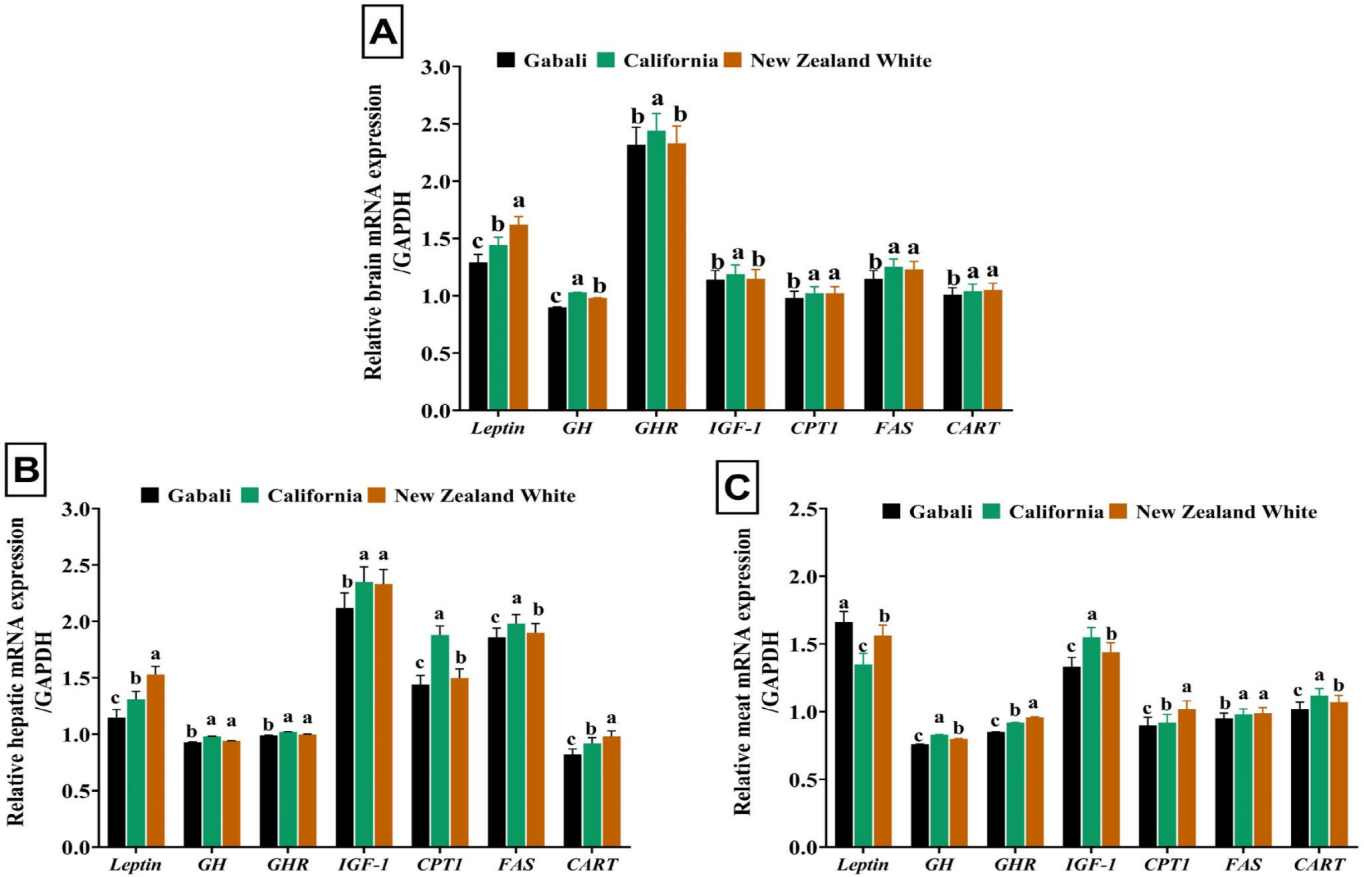
Gene expression profile of Leptin, *GH*,* GHR*,* IGF-1*,* CPT1*,* FAS*, and *CART* genes in brain (**A**), liver (**B**), and meat (**C**) tissues of California, New Zealand White, and Gabali rabbits. *CART*, cocaine-amphetamine-regulated transcript gene; *CPT1*, carnitine palmitoyltransferase 1 gene; *FAS*, fatty acid synthase gene; *GAPDH*, glyceraldehyde-3-phosphate dehydrogenase gene; *GH*, growth hormone gene; *GHR*, growth hormone receptor gene; *IGF-1*, insulin-like growth factor 1 gene;

Moving to Fig. 2, CAL rabbits exhibited the highest gene expression levels for insulin-like growth factor 1 (*IGF-1*), growth hormone (*GH*), and growth hormone receptor (*GHR*) genes in brain tissues, indicating potential molecular mechanisms underlying growth and development in this breed. In brain tissues, NZW rabbits displayed the highest gene expression profile for leptin, a hormone involved in energy balance and metabolism regulation. Conversely, liver tissue analysis showed no significant differences between CAL and NZW rabbits in the gene expression profiles of *GH*, *GHR*, and *IGF-1*. However, CAL rabbits exhibited clear upregulation in the gene expression profiles of carnitine palmitoyltransferase 1 (*CPT1*) and fatty acid synthase (FAS), while leptin maintained the highest expression levels in NZW rabbits. Additionally, minor variations in the gene expression profiles of *GH*, *GHR*, *CPT1*, *FAS*, and *CART* were observed in meat tissues, with *IGF-1* notably high in CAL rabbits and leptin exhibiting the highest expression in GAB rabbits.

## Discussion

In this study, Significant breed effects were observed in litter traits, with CAL and NZW rabbits exhibiting higher values compared to GAB rabbits, consistent with previous findings (Belabbas et al. 2023; Krupová et al. 2020; Montes-Vergara et al. 2021; Sosa-Madrid et al. 2020). Breed effects were also significant for LSB, LSW, LWB, and LWW (Meky and Altahawy 2023). Differences in LSB between breeds could be attributed to variations in uterine capacity, conception rate, established ova, and fertilization (Pinto-Pinho et al. 2023; Popli et al. 2022).

Breed significantly influenced BW traits at different ages, with CAL and NZW rabbits recording the highest values compared to GAB rabbits (Farouk et al. 2022; Palka et al. 2023). DG was also significantly influenced by breed, with CAL rabbits showing the highest estimates and GAB rabbits recording lower estimates (Fang et al. 2020; Krupová et al. 2020; El-Deghadi et al. 2023).

Heritability (h^2^) estimates for litter traits ranged from low to medium, consistent with previous studies (Adeolu et al. 2020; Ezzeroug et al. 2019; Rabie et al. 2020; Ramadan et al. 2020; Peiró et al. 2021). Similarly, moderate heritability estimates were observed for BW at different ages (Rabie et al. 2020; El-Deghadi et al. 2023; Farouk et al. 2022). These results suggest potential genetic influences on these traits, although non-genetic factors also play significant roles (Nguyen et al. 2021; Ghildiyal et al. 2023). The heritability results align with those reported in previous investigations for BW at different ages (Rabie et al. 2020; El-Deghadi et al. 2023; Farouk et al. 2022), indicating the potential for genetic selection to improve these traits in rabbit breeding programs. The moderate heritability estimates for body weight (BW) at 4, 8, and 12 weeks suggest that selective breeding can lead to improvements in body weight. Notably, the highest heritability estimate for body weight gain was observed during the 8–12 week period, while the lowest estimate was found for the 4–8 week period (Mínguez et al. 2015; Carballo et al. 2019; El-Deghadi et al. 2023). Differences in heritability estimates can be attributed to genetic variation among lines or breeds, estimation methods for environmental fluctuations, variance components, and data set sizes (Mínguez et al. 2016).

Positive and moderate genetic correlations (rg) among all litter sizes suggest that selecting for number of kits born alive (NBA) and litter size at birth (LSB) would lead to an increase in litter weight at birth (LWB), consistent with previous research (Ezzeroug et al. 2019; Farouk et al. 2022). Additionally, selecting for LWB, litter weight at 21 days (LW21d), and litter size at weaning (LSW) would contribute to the improvement of litter weight at weaning (LWW) as these traits were positively correlated (Ramadan et al. 2020; Hassan 2005). The phenotypic correlations between litter traits and between litter weight and body weight were consistent with previous studies (Egena et al. 2012; Belabbas et al. 2021; Shehab El-Din 2022). Furthermore, the genetic correlation between BW and DG was moderately to highly positive, suggesting that selecting rabbits with higher BW at an early age positively impacts their weight at marketing, as reported by Rabie et al. (2020) and El-Deghadi et al. (2023). The positive and moderate phenotypic correlations between different BW records and DG at different age stages provide insights for effective management (Croda-Andrade et al. 2022; Montes-Vergara et al. 2021; Sánchez et al. 2022). These findings emphasize the importance of understanding the genetic and phenotypic relationships between traits for the development of selection indices aimed at enhancing the performance of farm animals (Bangar et al. 2022; El-Attrouny and Habashy 2020).

Carcass traits at 12 weeks of age were significantly influenced by breed, with carcass weight, full gastrointestinal tract weight, hot carcass weight, heart weight, liver weight, and subcutaneous fat weight all showing significant genotype effects. These results are consistent with previous studies indicating breed effects on various carcass traits at different ages (Abd El-Aziz et al. 2022; Belabbas et al. 2019; North et al. 2019; Suleman et al. 2020). Additionally, factors such as litter size, parity, maternal effects, and environmental conditions play significant roles in influencing rabbit growth.

The gene expression profiles observed in this study offer insights into the molecular mechanisms underlying growth and metabolic processes in New Zealand White (NZW), Californian (CAL), and Gabali (GAB) rabbit breeds. NZW rabbits exhibited significantly higher expression levels of genes associated with energy metabolism, appetite regulation, nutrient transport, and G protein-coupled receptors in brain tissues compared to CAL and GAB rabbits (Yang et al. 2013, 2020; Landry et al. 2021; Agyekum et al. 2015; Fu et al. 2017). CAL rabbits showed elevated expression of the *ACC* gene in brain tissues, potentially indicating a role in fatty acid biosynthesis and energy metabolism (Catlin et al. 2021).

In liver tissues, CAL rabbits exhibited greater expression levels of genes regulating growth and development, including *IGF-1*, *GH*, and *GHR* genes, suggesting enhanced growth potential at the molecular level compared to NZW rabbits (Helal et al. 2022). However, no significant variations were observed in the expression patterns of certain genes related to fatty acid oxidation and lipogenesis between CAL and NZW rabbits in liver tissues (Liu et al. 2019).

In meat tissues, GAB rabbits demonstrated the highest expression levels of genes associated with energy metabolism, appetite regulation, and nutrient transport, suggesting unique molecular adaptations related to energy utilization and appetite regulation in GAB rabbits (*TBC1D1*, *ACC*, *NPY*, *AGRP*, *GPR*). These findings contribute to our understanding of the genetic factors influencing growth and metabolism in different rabbit breeds, providing valuable insights for future breeding and management strategies.

In summary, Significant breed effects were observed in litter traits, with CAL and NZW rabbits generally exhibiting higher values compared to GAB rabbits, which is consistent with previous findings. Breed also significantly influenced body weight traits at different ages, with CAL and NZW rabbits typically recording higher values compared to GAB rabbits. Moreover, carcass traits at 12 weeks of age were significantly influenced by breed, highlighting the importance of genetic factors in determining carcass characteristics.

The heritability estimates for litter traits and body weight were found to range from low to moderate, indicating potential genetic influences on these traits. Additionally, positive genetic correlations among litter sizes and between litter weight and body weight suggest opportunities for selection to improve these traits collectively. Furthermore, the gene expression profiles revealed unique molecular adaptations related to energy metabolism, appetite regulation, and nutrient transport in different tissues among the three rabbit breeds, providing valuable insights into the underlying mechanisms of growth and metabolism.

## Conclusion

This study investigated the breed effects on various traits in New Zealand White (NZW), Californian (CAL), and Gabali (GAB) rabbit breeds. Significant differences were observed in litter traits, body weight (BW) at different ages, daily gain, and carcass traits among the breeds. Heritability estimates indicated potential genetic influences on these traits, with moderate heritability observed for BW. Positive genetic correlations among litter sizes and between litter weight and body weight suggest potential for selective breeding. Gene expression analysis revealed distinct molecular mechanisms underlying growth and metabolism in the different breeds, with NZW, CAL, and GAB rabbits exhibiting unique genetic profiles related to energy metabolism and appetite regulation. These findings provide valuable insights for rabbit breeding and management strategies aimed at enhancing performance. Further research is needed to elucidate specific genetic pathways and mechanisms underlying these traits for targeted breeding efforts.

## Data Availability

The data that support the findings of this study are available from the corresponding author upon reasonable request.
